# Telehealth Emergency Department Transition-of-care Program: A Value-based Innovation

**DOI:** 10.5811/westjem.41524

**Published:** 2025-09-01

**Authors:** Allyson Kreshak, Itzik Fadlon, Karna Malaviya, Vaishal Tolia, Lindsey Pierce, Theodore Chan, Parag Agnihotri, Ming Tai-Seale

**Affiliations:** * University of California, San Diego Health, Population Health Services Organization, San Diego, California; † University of California, San Diego Health, Department of Emergency Medicine, San Diego, California; ‡ University of California, San Diego, Department of Economics, San Diego, California; § University of California, San Diego Health, Department of Family Medicine, San Diego, California; || University of California, San Diego Health, Department of Medicine, Division of Biomedical Informatics, San Diego, California

## Abstract

**Introduction:**

Our Emergency Department (ED) and Population Health Services Organization developed a telehealth ED-transition of care program (TOC) for patients insured through value-based contracts. This study’s goal was to determine the association of our ED-TOC on ED revisits. We hypothesized that the ED-TOC would decrease ED revisits.

**Methods:**

This was a retrospective cohort study conducted between August 1, 2021 and July 31, 2023 at two EDs where an ED-TOC is available. Included were ED visits among discharged Medicare beneficiaries that occurred one year before and after the launch of the ED-TOC program. ED visits involving Medicaid beneficiaries served as the control. A difference-in-differences (DID) strategy was used to compare Medicare and Medicaid visits. The primary outcome measure was the association of the program with 14- and 30- day ED revisit rates. Secondary outcomes were the association of the ED-TOC with post-discharge PCP visits and hospitalizations and estimated cost-savings associated with the program.

**Results:**

Our sample size was 23,696 ED encounters (13,553 treatment group and 10,143 control group). At 14-days after ED discharge, Medicare beneficiaries were associated with a 1.77% decrease in the rate of ED revisits in the year after the ED-TOC launch relative to the control (p=0.03) or a 15.8% reduction relative to baseline (11.2% to 9.4%). At 14-days after ED discharge, PCP visits were associated with a 1.51% increase in the year after program launch relative to the control (p=0.03) or a 10.3% increase relative to baseline (14.6% to 16.1%). No difference was associated with Medicare beneficiaries’ ED revisits or hospitalizations at 30-days. PCP visits were associated with a significant increase at 30-days (p=0.005).

**Conclusion:**

An ED-TOC is associated with a reduction in Medicare ED revisits during days 8–14 after an index ED visit but not during days 1–7 days or at 30-days. Cost savings over a 24-week period are conservatively calculated to be $215,779.

## INTRODUCTION

Emergency department (ED) follow-up care after discharge may ensure that a patient’s health issues addressed in the ED are appropriately managed. In the United States in 2021, an estimated 140 million ED visits occurred, where 75% were dispositioned to outpatient follow-up.^1^ Adults ≥ 65 years of age comprise 18% of these visits, and 65% of this age group are discharged to home.^2^ ED care transitions for older adults are rife with limited patient understanding of their clinical condition and suboptimal communication.^3^ Complicating the situation, ED discharge instructions are poorly understood with limited rates of adherence to prescription medications, follow-up plans, and reasons to return to the ED.^4^ Not surprisingly, ED revisits occur due to care issues relating to diagnosis, treatment, disease progression, challenges with securing follow-up care, non-compliance, and imperfect communication during the ED encounter.^6^–^10^ The percentage of patients who revisit the ED varies widely with estimates of up to 7.5% at 72 hours, 8.5% within seven days, 11.5% within 14 days, and 20% within 30 days.^11^,^12^ These ED revisits lead to increased costs, increased ED patient volumes, mortality, and treatment delays.^13^

Prevention of ED revisits and enhancing quality of care is a focus of ED transition-of-care programs (TOC).^14^ Models of ED-TOCs largely consist of telephone follow-up and have had varied outcomes. Two ED-TOCs feature nurse callbacks^15^ and automated callbacks with the option to speak with an advanced practice clinician;^13^ both programs resulted in decreased ED revisits at seven days. Additionally, an ED-TOC involving consultation with an ED nurse was associated with decreased Medicare expenditures.^16^ Other ED-TOCs with non-physician practitioner telephone follow-up, however, did not impact ED revisits at 30 days.^17^–^19^ Additionally, ED follow-up visits with primary care physicians (PCP) conducted via telehealth were associated with higher ED revisits at 30 days compared to in-person visits.^20^

The potential of these ED-TOCs to influence a patient’s care is important for improving quality of care and for addressing broader healthcare challenges. Emergency departments are experiencing increases in patient volume and acuity,^2^,^21^,^22^ attributed largely to a growing and aging population and inadequate access to care,^4^,^22^ while also facing crowding and boarding issues.^23^,^24^ Additionally, hospitals face rising costs, inpatient capacity constraints, and staff shortages.^25^,^26^ Programs that safely transition patients out of the ED have the potential to mitigate ED revisits and potentially hospitalizations and to enhance care management.^14^ The previously described ED-TOCs with mixed ED-revisit impact feature different modalities for ED follow-up and involve nurses, paramedics, and PCPs. The ideal training level of the practitioner providing TOC services remains in question as does the best TOC system. Our ED-TOC builds upon this work and provides emergency physician-led telehealth visits coupled with robust care navigation to eligible patients discharged from the ED.

The purpose of this study was to determine the association of our ED-TOC program involving emergency physician-led telehealth visits and robust care navigation with ED revisits.

## METHODS

This quality improvement project was deemed non-human subjects research and exempt from institutional review board review by the University of California San Diego Aligning and Coordinating Quality Improvement, Research and Evaluation Committee.

**Population Health Research Capsule**

What do we already know about this issue?

*Revisits to the ED are associated with increased costs, mortality, and treatment delays. Previous models of ED transitions of care (TOC) have had varied impact on revisits*.

What was the research question?

*What is the association of an ED-TOC program involving physician telehealth visits and care navigation with ED revisits?*

What was the major finding of the study?

*Medicare patients were associated with a 1.77% drop in the ED revisit rate, or a 16% drop in ED revisits (P = .03)*.

How does this improve population health?

*An ED-TOC program with accountability for cost and quality may enhance care by decreasing the number of ED revisits*.

### Study Design and Setting

In this retrospective cohort study we used data from two EDs in an academic health system: ED1 is a suburban, quaternary-care hospital ED; and ED2 is an urban hospital ED. The combined census is 89,000 patients per year with a discharge rate of 76%. Our health system’s Population Health Services Organization (PHSO) is a team focused on improving the health of specific populations through data analysis, targeted interventions, and collaboration with clinicians and community partners to achieve better health outcomes and reduce healthcare costs. The PHSO is the delegated entity for health plan oversight for our health system’s attributed health maintenance organization (HMO) patients, specifically commercial and Medicare Advantage plans. The PHSO is responsible for delivering high-quality, cost-effective care (ie, value-based care) for these insured patients. In this model, fixed payments per patient per month are available to the PHSO regardless of the number of services provided, and quality standards are established. Medicaid contracts are not part of the PHSO. The percentage of patients attributed to the PHSO is approximately 11% at ED1 and 7% at ED2.

### Selection of Participants

Included in this study were beneficiaries covered by Medicare Advantage plans, traditional fee-for-service Medicare, and Medi-Cal (California’s Medicaid program) who were discharged from either ED in the year before and year after the launch of the ED-TOC program. Traditional Medicare patients were included given that the Centers for Medicare & Medicaid Services (CMS), in a recent report, tied 90% of traditional fee-for-service Medicare payments to value.^27^ Importantly, this focus on value incentivizes health systems and clinicians to concentrate on care quality and cost-efficiency rather than just volume of services provided.

### Intervention

In July 2022, our ED-TOC was established. This program is a collaboration between our ED and our health system’s PHSO. Patients eligible for the ED-TOC are those PHSO-attributed ED patients and patients insured with traditional fee-for-service Medicare who are discharged from the ED. The ED-TOC staff contacts patients in the ED for participation and enrollment in the transition-of-care program or within 72 hours after ED discharge. Within 24 hours of enrollment, patients receive a telehealth visit with one of our ED-TOC physicians (an emergency physician designated to this role). The ED-TOC physicians underwent eight hours of combined telehealth training and program-specific training prior to participation in the program. The telehealth visit addresses the patient’s acute medical issue for which s/he presented to the ED.

Depending on clinical needs, which are determined while the patient is in the ED or during the ED-TOC telehealth visits, patients may also receive home-health nursing, remote patient monitoring, in-home parenteral medications, in-home diagnostic studies (electrocardiogram, radiographs, ultrasound), physical therapy, wound care, and phlebotomy, if medically indicated. Patients remain active with the program until acute medical needs have stabilized or resolved. Inherent to the program is a robust care navigation team that assists every enrolled patient. The ED-TOC team schedules follow-up PCP appointments prior to the patients detaching from the program. Patients do not need to have an established follow-up PCP appointment prior to enrollment in the ED-TOC. The median length of stay within the ED-TOC is five days.

### Measurements

For each ED encounter, we created a dataset of the 24 weeks that followed index ED discharges. We merged in the following information: ED visits; PCP visits; and inpatient hospitalizations. Data extraction was performed by professional staff in Information Services who were not blinded to the goals of the ED-TOC program.

Discharges from the ED in the year prior to program launch were defined “pre-period” (August 1, 2021–July 25, 2022), and those after the program’s start were defined “post-period” (July 26, 2022–July 31, 2023), so that observations were split exactly around the launch date. For each patient we kept only the first encounter within our data range to limit omitted-variable bias, as the program may influence the composition of cases associated with revisits. This restriction also reduced the risk of contamination, whereby outcomes at an encounter may have reflected the ongoing impacts of a previous encounter. By construction, this implies that more patient encounters were included in the pre-period (65% of analyzed encounters).

We estimated the association between the ED-TOC and ED revisits, PCP visits, and hospitalizations using a difference-in-differences (DID) strategy, which is widely used in social science research.^28^ The DID approach is an established method in clinical research for estimating the impact of an intervention or program when randomized trials are not feasible, such as in clinical settings where interventions are present in some groups but not others. The DID compares changes in established outcomes over time between a treatment and comparable control group to assess whether the intervention really had an impact despite other background changing conditions. Outcomes in both the treatment and control groups are measured before the intervention and after the intervention, and calculations are done to determine how much each group has changed over time. The DID methodology works with the assumption that, in the absence of the studied intervention, the treatment and control groups would have followed similar trends over time. This is known as the parallel trends assumption.

The treatment group was composed of traditional Medicare and Medicare Advantage patients who were eligible for the ED-TOC in the post-period. To address the challenge posed by general time trends unrelated to the program during the study period, we used Medi-Cal patients, whose participation in the ED-TOC was effectively null because they were not offered the program, as a control group. Although traditional Medicare and Medicare Advantage patients differ from Medi-Cal patients in demographics and potentially in the levels of outcomes of interest, DID methodology allows for such persistent levels of differences between treatment and control. The key assumption is that the change in outcomes experienced by Medi-Cal beneficiaries from the pre-period to the post-period approximated the change in outcomes that traditional Medicare and Medicare Advantage beneficiaries would have experienced if the ED-TOC program had not launched.

**Figure 1:**
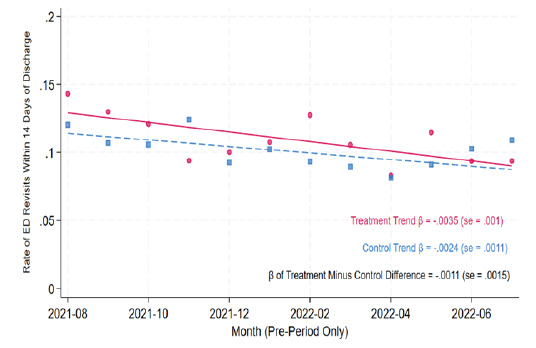
Time trends across the treatment and control groups regarding 14-day emergency department revisit rates. Note: Points represent the average rate of ED visits in days 1–14 after discharge among Medicare/Medicare Advantage patients (treatment group) and Medi-Cal patients (control group) discharged in a given calendar month. The solid and dashed lines plot time trends for the treatment group and the control group, respectively, calculated with a patient-level linear regression of ED visits 1–14 days after discharge on month of discharge. The difference in slopes between treatment and control was calculated from a linear regression of ED visits 1–14 days after discharge-on-discharge month, a treatment-group indicator variable, and discharge month interacted with the treatment-group indicator variable. Robust standard errors are clustered at the patient level. ED, emergency department; Medi-Cal, California Medicaid.

The assumption required for DID methodology to work for this analysis is that 14-day ED revisits for Medicare/Medicare Advantage and Medi-Cal follow similar trends over time. The validity of this assumption was tested in Figure 1 with the standard parallel pre-trends test that studied the similarity of monthly average pre-period time trends across the treatment and control groups regarding 14-day ED revisit rates. The sample included 13,553 traditional Medicare and Medicare Advantage encounters and 10,143 Medi-Cal encounters, and trends were based on a linear regression in calendar time in months (estimated on encounter-level data). The time patterns in 14-day ED revisit rates across the two groups showed no secular differences between them, thereby supporting the use of DID methodology for this analysis.

### Outcomes

Our primary outcome measure was 14- and 30-day ED revisits. Secondary outcome measures were post-ED PCP visit rates and inpatient hospitalization rates up to 30 days after the index ED visit.

### Analysis

Our DID methodology captured the differential change over time between the treatment and control groups, isolating the effect attributable to the ED-TOC under the parallel trends assumption described above. Conceptually, this methodology compared two changes. First, the “treatment difference” was the change in outcomes from the pre- to post-implementation period in the treatment group—patients eligible for ED-TOC enrollment. This change captured both the intervention’s effect and any temporal trends unrelated to it. Second, the “control difference” was similarly defined as the pre- to post-implementation change in outcomes among the control group—patients ineligible for the ED-TOC—capturing underlying temporal trends unrelated to the program. Finally, we calculated the DID estimate of the program’s effect as the treatment difference minus the control difference, isolating the portion of changes over time that could be attributed to implementation of the ED-TOC.

We interpreted our DID estimates as intent-to-treat effects because treatment group assignment was based on eligibility for the ED-TOC, regardless of whether patients chose to enroll. Specifically, our findings reflect the impacts of offering the program to patients rather than the impacts of enrollment. This is the most program-relevant interpretation because, in practice, the program was available to eligible patients without mandating participation. To examine the program’s impact over time, we calculated separate DID estimates for each consecutive 14-day period after ED discharge up to 24 post-discharge weeks. Given previously reported rates of ED revisits^11^,^12^ and conventional reporting standards, we also calculated DID estimates for days 1–7, days 8–14, and for the full 30-day period after ED discharge.

To support clinical interpretation, we present our DID estimates of the ED-TOC program’s effects in three complementary forms. First, we report the change in outcome rates associated with the program: These are our raw DID estimates, representing the change in the percentage of discharges that resulted in ED revisits, PCP visits, and inpatient hospitalizations following ED-TOC implementation. Second, we express the effects as a percentage change relative to baseline by dividing the raw DID estimates by the treatment group’s pre-period mean. Third, we estimate the per-enrollee effect by dividing the raw DID estimate by the ED-TOC enrollment rate, approximating the change in outcomes among patients who participated in the program.

Statistical details of our DID regression analysis are described in the Appendix. All regressions controlled for seasonal trends and derived *P*-values from standard errors clustered at the patient level. To assess the sensitivity of our findings, we evaluated the stability of estimates with and without covariate adjustment for observed patient characteristics.

## RESULTS

### Summary Statistics

Our sample size was 23,696 ED encounters (Table 1). Differences in observable characteristics between treatment and control did not threaten our DID strategy, which compared changes in outcomes before and after program launch within each group. Furthermore, our main results were robust to controlling for these observable characteristics (see Appendix Table 2).

### Main Results

**Figure 2:**
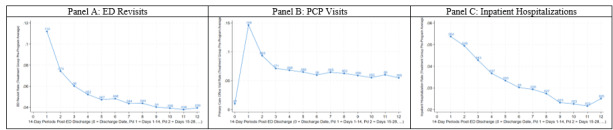
Outcomes following emergency department visit (14-day frequency)—baseline dynamics. Note: This figure displays mean outcomes in 14-day periods since ED discharge among Medicare and Medicare Advantage patients (the treatment group) in the 12 months prior to launch of the ED TOC program. *ED*, emergency department; *PCP*, primary care physician; *TOC*, transition of care.

Table 2 details the effects associated with the ED-TOC program in days 1–14 and 15–28 after discharge. For clinical interpretation, the table reports the change in outcome rates after program implementation (ie, the DID estimate) and the percentage change in outcomes relative to baseline for each primary and secondary outcome. Figure 2 provides the treatment group’s baseline outcomes after discharge in the pre-period. In the year after program implementation, 7.03% of treatment-group patients enrolled in the ED-TOC within 14 days after ED discharge (*P* < .001). Enrollment declined thereafter, consistent with the ED-TOC protocol.

We found that the program was associated with a 1.77% reduction in ED revisit rates in the treatment group relative to the control group in the 14-day period after discharge (*P* = .03). The decline is statistically significant in days 8–14 after discharge (−1.35%, *P* = .02) and smaller and statistically insignificant in 1–7 days after discharge (−0.67%, *P* = .34). No significant change in the ED revisit rate occurred during days 15–28.

To express the decline in ED revisit rates as a percentage change relative to baseline, we divided the DID estimates by the treatment group’s baseline pre-period ED revisit rate (Figure 2). This analysis shows that the program was associated with a 15.8% (−1.77%/11.2%) change relative to baseline in days 1–14 after discharge or equivalent to a decline from 11.2% to 9.4%. Dividing the ED revisit rate by the ED-TOC enrollment rate approximates the effect per enrollee, indicating that the program was associated with preventing the 14-day ED revisits among 25.2% (−1.77%/7.03%) of enrollees.

Further, the program was associated with an equal-sized, opposite effect for PCP visit rates (Table 2). The results indicate an increase in the rate of PCP visits at both 1–7 days after discharge (0.76%, *P* = .20) and 8–14 days after discharge (0.33%, *P* = .52), although neither result is statistically significant. When considering days 1–14 after ED discharge, however, the increase (1.51%) was statistically significant (*P* = .03). There continued to be an increase in PCP visits at days 15–28 (*P* = .20). Relative to the baseline PCP visit rate, we found that the ED-TOC was associated with a 10.3% increase (1.51%/14.6%) in 14-day PCP visits, equivalent to an increase from 14.6% to 16.1%. Dividing the PCP visit rate by the ED-TOC enrollment rate approximates the effect per enrollee, indicating that the program led to 14-day PCP visits for 21.5% (1.51%/7.03%) of enrollees. There were no statistically significant effects on inpatient hospitalization rates during days 1–14 and 15–28 after discharge (Table 2).

Appendix Table 1 provides outcomes for the 30-day period following ED discharge. We found an associated 1.31% decline in ED revisit rates in the 30-days following discharge (*P* = .18); a 2.32% rise in PCP visit rates (*P* = .005); and insignificant associations with 30-day hospitalizations (*P* = .93).

Appendix Table 2 adds a vector of covariates to account for compositional differences across groups, showing the robustness of our results to their inclusion. The covariates include age at discharge, age at discharge squared, sex, and indicators for race/ethnicity.

## DISCUSSION

The findings of this study demonstrate that a physician-staffed, telehealth ED-TOC was associated with a 16% relative reduction in the ED revisit rate for Medicare beneficiaries up to two weeks after ED discharge and a 10% increase in visits to PCPs at 14 days after ED discharge.

When Medicare was considered the primary expected payor for an ED visit, this decline in ED visits translated to roughly $252,584 in cost savings during our 24-week study period if a historical cost of $1,040 per Medicare ED treat-and-release visit is referenced^29^ and a baseline ED revisit rate of 11% at two weeks is used. When factoring the increased PCP visits and related costs ($36,805),^30^ the net savings over the study period (24 weeks) was calculated at $215,779 within the treatment group. These values are conservative given the annual growth in ED mean costs^31^ and that this study was conducted during the program’s first year of implementation when workflows were new and developing efficiency. The annual investment in the program was $1,400,000. The main expenses are the physician and supporting staff (nurse program manager, nurse case manager, and medical assistant) costs. From its inception, the program has leveraged the health system’s established electronic health record and telehealth processes and protocols. The potential cost-savings and health benefits of reduced ED revisits^13^ bring value to this type of program for organizations working with capitated health plans (eg, HMOs) in which a fixed payment per patient per month is available. Additionally, the harder-to-quantify benefits are in reduced ED patient volume in EDs that are already facing crowding and boarding issues.^23^,^24^

The association of our program with the ED revisit rate was assessed for up to 30 days. We did not examine the 72-hour revisit rate because patient enrollment in the program occurred up to 72 hours after ED discharge. We expected that the 1–7 day ED revisit rate among Medicare patients would have decreased. Interestingly, though, there was no difference in ED revisits up to seven days in the Medicare group when compared to the control group. Instead, the decline in ED revisits associated with our program occurred between 8–14 days. These findings align with the findings of Rising et al who suggested that a nine-day ED return visit rate is a more appropriate time frame to capture early ED return visits related to the initial ED visit rather than a 72-hour or 7-day return revisit timeframe.^32^ In this regard, our use of a 14-day ED revisit rate would meaningfully capture potential early ED revisits.

In addition to reducing ED revisits, our data suggest that our ED-TOC is also associated with enhanced access to PCPs after an ED visit. These findings are consistent with other ED follow-up programs. Luciani et al^15^ showed that implementation of nurse phone calls at 24–96 hours after ED discharge resulted in a 4.1% decrease in ED revisits and a 17% increase in 7-day follow-up PCP appointments. Fruhan and Bills^13^ studied patients who received an automated phone call at two days after ED discharge with the option to connect with an advanced practice clinician. At seven days post-discharge, they reported a decrease in ED revisits among patients who had ≤ 3 ED visits in the 180 days preceding the index ED visit.

Jacobsohn et al^19^ had paramedics conduct in-home patient visits 24–72 hours after ED discharge with subsequent coaching phones calls. They reported no change in ED revisits at 14 or 30 days but increased outpatient follow-up within one week after ED discharge. Hastings et al^18^ demonstrated that ED patients who received post-ED discharge calls up to 1–2 weeks after ED discharge had a higher rate of PCP visits at 30 days but no change in 30-day ED revisits. Biese et al^17^ studied the impact of nurse-led telephone calls after ED discharge. These follow-up calls did not reduce 30-day ED revisits or hospitalizations. Furthermore, a comparison by Shah et al^20^ of in-person vs telehealth PCP follow-up visits within 14 days of ED discharge to home revealed a higher rate of ED revisits and hospitalizations among the telehealth visit group, thereby suggesting the need for additional investigation into ED telehealth follow-up. Our ED-TOC with daily physician telehealth visits was associated with a higher increase in PCP follow-up and a higher decrease in early ED revisit rates than these previous studies. We attribute the drop in ED revisits and increased access to PCPs to the ED-TOC intervention in addressing patient diagnoses and response to treatment plans, patient adherence, and care navigation—factors that drive patients to return to the ED.^6^–^10^,^33^

The lack of effect of this ED-TOC on ED return visits beyond two weeks is reflective of the medically complex, quaternary-care population that our EDs serve. The overall ED admission rate of our two EDs is 24.5%, reflecting the higher acuity of the patient population compared to EDs with a similar census at 15% and similar type at 12%.^34^ The implementation of other initiatives referenced in the literature may help reduce ED return visits further. These programs include the availability of telehealth for acute unscheduled visits, pre-ED telehealth triage, and engaging patients who are frequent ED users with a care plan.^35^

As the population of aging adults with complex care issues continues to grow, demand for ED services will also increase. Roughly 65% of older adults are discharged to home from the ED,^2^ but the medical issues that cause older patients to seek care in the ED have been associated with a decline in functional status.^36^ Historically, ED discharge has been accompanied by limited communication and understanding of care needs.^3^ The availability of ED value-based programs, such as a geriatric ED and an ED-TOC, allows for patient care needs to be identified and outpatient resources accessed.^14^,^16^,^37^ In this way, ED visits become further integrated into the care continuum. Currently, Medicare is the primary payor for 87% of patients ≥ 65 years of age,^2^ and approximately 47% of Medicare beneficiaries are covered by Medicare Advantage. With the CMS 2030 goal of having all fee-for-service Medicare beneficiaries engaged in a care program with accountability for quality and total cost of care, innovative value-based programs are becoming increasingly important.^38^ Additionally, the ability of a TOC program to decrease ED revisit rates and improve access to outpatient care is important to hospitals given the increasing rates of ED visits nationwide^2^,^14^ and the limited inpatient capacity of hospitals, which together contribute to ED boarding and long ED wait times.^23^,^25^,^39^

Under the traditional Medicare fee-for-service model, an ED-TOC such as ours would be costly and difficult to sustain, as reimbursement for these TOC services for a visit originating in the ED is not directly supported.^40^ Our ED-TOC represents a partnership between the ED and our health system’s PHSO, which manages the care of patients insured through HMOs. When part of a care relationship involving accountability for total cost of care and quality, an ED-TOC becomes a valuable innovation and potential cost-saver through its ability to decrease ED revisits and enhance access to care. With the fragile fee-for-service payment model currently in place for US EDs,^35^ these value-based programs involving ED care may become increasingly important.

## LIMITATIONS

This study was conducted at a single health system with an integrated PHSO; therefore, results may not be applicable to other health systems. Because this was a quality improvement project not designed as a randomized controlled trial, causality could not be determined. The results of the DID methodology suggest causation but rely on the assumption that the control group before-and-after change approximates the treatment group before-and-after change in the absence of the TOC program. While the Medicare and Medi-Cal populations may differ in observable characteristics, Figure 1 shows parallel time trends between the groups in the pre-period. However, we cannot rule out the possibility that factors unrelated to the TOC program, such as economic changes, contributed to differential before-and-after changes for treatment and control. Restricting our sample to each patient’s first encounter limited the interpretation of our results to capture the program’s impact on initial ED encounters only. While this restriction implies that the post period mechanically contains fewer patients who frequently visit the ED, this factor acts upon both the treatment and control groups and did not pose a direct threat to our DID design.

## CONCLUSION

An ED telehealth transition of care program was associated with a reduction in Medicare ED revisits to the emergency department during days 8–14 after an index ED visit but not during days 1–7 or at 30 days. Visits to primary care physicians visits were associated with a significant increase at 14 and 30 days. No association was found with 30-day hospitalizations.

## Supplementary Information





## Figures and Tables

**Table 1:** Patient characteristics for analysis sample of 23,696 emergency department encounters.

PHSO patient ED encounters	Experimental group	

	Control, n (%)	Treatment, n (%)
	10,143 (42.8%)	13,553 (57.2%)
Sex		
Male	4,679 (46.1%)	6,156 (45.4%)
Female	5,459 (53.8%)	7,396 (54.6%)
Other	5 (0.0%)	1 (0.0%)
Race/Ethnicity		
Non-Hispanic White	3,127 (30.8%)	8,019 (59.2%)
Hispanic	3,573 (35.2%)	2,195 (16.2%)
Black	1,499 (14.8%)	875 (6.5%)
Asian	541 (5.3%)	1,390 (10.3%)
Other	1,403 (13.8%)	1,074 (7.9%)
Mean age in years (SD)	45.0 (16.3)	68.9 (16.3)
< 65 years	9,207 (90.8%)	3,539 (26.1%)
> 65 years	936 (9.2%)	10,014 (73.9%)

Note: This table summarizes the characteristics of patients whose ED discharge outcomes were studied in our regression analysis. Patients were included in our analysis if they were insured by Medicare or Medicare Advantage (treatment group) and were eligible for Population Health Services Organization (PHSO) services, insured by or Medi-Cal (control group), were eligible for PHSO services, and were discharged from either of the university health system’s two EDs between August 1, 2021–July 31, 2023. We only considered each patient’s first ED discharge within the study period.

*PHSO*, Population Health Services Organization; *ED*, emergency department.

**Table 2:** Estimated difference-in-differences effects of the ED transition-of-care program on outcomes in the four weeks After ED discharge.

Days after ED discharge	% Program enrollment rate	ED revisits		PCP visits		Inpatient hospitalizations	
		
		Outcome rate change (%)	% Change relative to baseline	Outcome Rate Change (%)	% Change relative to baseline	Outcome rate cange (%)	% Change relative to baseline
Day 1–14	7.03	−1.77	−15.8	1.51	10.3	−0.40	−7.4
	(P < .001)	(P = .03)		(P = .03)		(P = .74)	
	[6.17,7.89]	[−3.39,−0.14]		[0.12,2.90]		[−2.79,1.99]	
Day 1–7	6.88	−0.67	−8.8	0.76	8.7	−0.12	−3.3
	(P < .001)	(P = .34)		(P = .20)		(P = .92)	
	[6.03,7.72]	[−2.05, 0.71]		[−0.39,1.92]		[−2.49,2.25]	
Day 8–14	2.13	−1.35	−22.1	0.33	4.9	−0.05	−1.3
	(P < .001)	(P = .02)		(P = .52)		(P = .97)	
	[1.70,2.57]	[−2.51,−0.18]		[−0.68,1.34]		[−2.41,2.30]	
Day 15–28	1.55	0.30	4.1	0.76	8.2	0.65	13.3
	(P < .001)	(P = .66)		(P = .20)		(P = .59)	
	[1.17,1.93]	[−1.01,1.61]		[−0.40,1.92]		[−1.71,3.00]	

Note: This table reports our difference-in-differences (DID) estimates of the ED-TOC program’s effects, capturing the differential change in primary and secondary outcomes across the pre- and post-implementation periods between the treatment and control groups. Under the parallel trends assumption, which implies that the control group’s change across the pre- and post-period captures temporal trends unrelated to the program, the DID estimates isolate the effects attributable to the ED-TOC. The “% program enrollment rate” column reports DID estimates of the ED-TOC enrollment rate among treatment group patients in the year after program implementation. For each primary and secondary outcome, the “outcome rate change” column represents our DID estimates, representing the change in percentage of ED discharges that resulted in the outcome of interest following program implementation. P-values and 95% confidence intervals are presented below each DID estimate in parentheses and brackets, respectively. The “% change relative to baseline” column expresses the DID estimate as a percentage change relative to baseline by dividing it by the treatment group’s baseline pre-period mean. The treatment group’s baseline pre-period mean outcomes for days 1–14 and 15–28 after ED discharge are shown in Figure 2.

*DID*, difference-in-differences; *ED*, emergency department; *TOC*, transition of care; *PCP*, primary care physician
