# Supplementary material for: Telehealth Emergency Department Transition-of-care Program: A Value-based Innovation
Source: West J Emerg Med. 2025 Sep 1;26(5):1202–10. doi: 10.5811/westjem.41524 (PMC12591659; doi:10.5811/westjem.41524)
Supplement: Supplementary file 2 [file wjem-26-1202-s002.docx]

**Appendix Table 2. Estimated DID Effects of the ED-TOC Program on Outcomes in the 4 Weeks After ED Discharge, With Demographic Controls**

|  |  | **ED Revisits** | | | **PCP Visits** | | | **Inpatient Hospitalizations** | | |  |
| --- | --- | --- | --- | --- | --- | --- | --- | --- | --- | --- | --- |
| Days After ED Discharge | % Program Enrollment Rate | Outcome Rate Change (%) | | % Change Relative to Baseline | Outcome Rate Change (%) | | % Change Relative to Baseline | Outcome Rate Change (%) | | % Change Relative to Baseline | |
| Day 1-14 | 7.05 | -1.70 | | -15.2 | 1.69 | | 11.6 | -0.76 | | -14.1 | |
|  | (p<0.001) | (p=0.04) | |  | (p=0.02) | |  | (p=0.53) | |  | |
|  | [6.19,7.90] | [-3.32,-0.07] | |  | [0.30,3.07] | |  | [-3.13,1.62] | |  | |
| Day 1-7 | 6.89 | -0.64 | | -8.4 | 0.87 | | 10.0 | -0.52 | | -14.4 | |
|  | (p<0.001) | (p=0.36) | |  | (p=0.14) | |  | (p=0.67) | |  | |
|  | [6.04,7.74] | [-2.02, 0.74] | |  | [-0.28,2.03] | |  | [-2.86,1.83] | |  | |
| Day 8-14 | 2.13 | -1.26 | | -20.7 | 0.40 | | 5.9 | -0.41 | | -10.3 | |
|  | (p<0.001) | (p=0.03) |  | | (p=0.44) |  | | (p=0.73) |  | |  |
|  | [1.70,2.57] | [-2.43,-0.10] |  | | [-0.61,1.41] |  | | [-2.74,1.92] |  | |  |
| Day 15-28 | 1.55 | 0.38 | 5.1 | | 0.83 | 8.9 | | 0.31 | 6.3 | |  |
|  | (p<0.001) | (p=0.57) |  | | (p=0.16) |  | | (p=0.79) |  | |  |
|  | [1.17,1.93] | [-0.93,1.69] |  | | [-0.33,1.99] |  | | [-2.-2,2.65] |  | |  |
| Day 1-30 | 7.21 | -1.17 | -7.3 | | 2.55 | 11.5 | | -0.41 | -4.9 | |  |
|  | (p<0.001) | (p=0.22) |  | | (p=0.002) |  | | (p=0.74) |  | |  |
|  | [6.34,8.07] | [-3.06, 0.72] |  | | [0.94,4.15] |  | | [-2.84,2.01] |  | |  |

Notes: This table reports our difference-in-differences (DID) estimates of the ED-TOC program’s effects when using covariate adjustment to account for compositional differences across the treatment and control groups. Under the parallel trends assumption, which implies that the control group’s change across the pre- and post-period captures temporal trends unrelated to the program, the DID estimates isolate the effects attributable to the ED-TOC. The “% program enrollment rate” column reports DID estimates of the rate of enrollment in the ED-TOC among treatment group patients in the year after program implementation. For each primary and secondary outcome, the “outcome rate change” column represents the DID estimates, representing the change in percentage of ED discharges that resulted in the outcome of interest following program implementation. P-values and 95% confidence intervals are presented below each DID estimate in parentheses and brackets, respectively. The “% change relative to baseline” column expresses the DID estimates as a percent change relative to baseline by dividing it by the treatment group’s baseline pre-period mean. The treatment group’s baseline pre-period mean outcomes for days 1-14 and 15-28 after ED discharge are shown in Figure 2.
